# Safety and efficacy of plasma exchange for the treatment of optic neuritis in neuromyelitis optica spectrum disorders: A protocol for systematic review and meta-analysis

**DOI:** 10.1097/MD.0000000000021067

**Published:** 2020-07-10

**Authors:** Mengyu Han, You Chen, Luqi Nong, Ziqiang Liu, Lu Hao, Zhijun Wang

**Affiliations:** a Science and education Department, Shenzhen Baoan Shiyan People's Hospital, Shenzhen; b Graduate School, Beijing University of Chinese Medicine; c Department of Ophthalmology, China-Japan Friendship Hospital, Beijing, China.

**Keywords:** neuromyelitis optica spectrum disorders, plasma exchange, protocol, systematic review

## Abstract

**Background:**

Neuromyelitis optica spectrum disorders (NMOSD) is an inflammatory and heterogeneous astrocyte disorder of the central nervous system (CNS), concerned because of its high pathogenicity, high risk of recurrence, and poor prognosis. Optic neuritis (ON) is the first manifestation in 30% to 50% of NMOSD patients, and eventually involved optic nerve in 70% of patients. The idiopathic ON associated with NMO is called NMO-associated ON(NMO-ON). There are substantial costs to the countries and individuals associated with treatment of NMO-ON. Intravenous corticosteroids (IVCSs), as the first-line therapy, leads to unsatisfactory outcomes for NMO-ON and is associated with potential adverse events (AEs). Emerging evidences have proved the important value and potential prospect of plasma exchange (PLEX) in NMO-ON. Although PLEX is increasingly used in NMO-ON, its therapeutic effect and safety are still controversial. There are no systematic reviews yet that evaluated the effects of PLEX against other therapies in patients with NMO-NO. It is therefore timely to perform a systematic review to assess the efficacy and safety of PLEX on current research for its potential use in clinical practice in treating NMO-ON.

**Methods:**

The systematic review will include all of the randomized controlled trials (RCT) on the efficacy and safety of PLEX for NMO-ON. A relevant literature search by sensitive search strategies was conducted using the following electronic databases from their inception to November 30, 2019: PubMed, Web of Science, EMBASE, the Cochrane Library, China National Knowledge Infrastructure (CNKI), Wanfang Database, China Science and Technology Journal database (VIP) and CBM. We will also search registers of clinical trials, potential gray literature, and conference abstracts. There are no limits on language and publication status. The literature screening, data extraction, and quality assessment will be conducted by 2 reviewers independently. The reporting quality and risk of bias will be assessed by other 2 researchers. Best-corrected visual acuity (BCVA), annualized relapse rate (ARR), the frequency and extent of adverse events (AEs) will be evaluated as the primary outcome. The secondary outcomes will include expanded disability status scales (EDSS), relapse-free rate, peri-papillary retinal nerve fibers layer (pRNFL) or macular volume, visual electrophysiology examinations, standard automated perimetry examinations, time to the next attack. Meta-analysis will be performed using RevMan5.3 software provided by the Cochrane Collaboration and Stata 12.0.

**Results:**

This study will provide a comprehensive review based on current evidence of PLEX treatment for NMO-ON in several aspects, including BCVA, ARR, the frequency and extent of adverse events (AEs), EDSS, relapse-free rate, etc.

**Conclusion:**

The conclusion of this study will provide evidence to determine whether PLEX is an effective and safe intervention for patients with NMO-ON.

**Ethics and dissemination:**

It is not necessary to obtain ethical approval for this study, given that this protocol is for a systematic review. The systematic review will be published in a peer-reviewed journal, presented at conferences and will be shared on social media platforms.

**PROSPERO registration number:**

PROSPERO CRD 42020162585.

## 1 Introduction

Neuromyelitis optica (NMO), also known as Devic disease, is currently considered to be a rare autoimmune astrocyte disease of the central nervous system mediated by autoantibodies, with optic neuritis(ON) and acute transverse myelitis as typical clinical manifestations.^[1]^ NMO has been recognized as a subtype of multiple sclerosis (MS) for more than 100 years since it was first described and reported.^[2]^ Until 2004, the discovery and confirmation of anti-aquaporin-4 immunoglobulin G (AQP4-IgG) made significant progress in pathogenesis, diagnosis, and treatment of NMO.^[3–4]^ The concept of neuromyelitis optica spectrum disorders (NMOSD) was first proposed based on the widespread clinical use of specific AQP4-IgG,^[4]^ which mainly referred to the limited NMO of positive AQP4-IgG. In 2015, the international NMO diagnostic team proposed a new international diagnostic standard for NMOSD.^[5]^ NMOSD includes NMO, ON, longitudinally extensive transverse myelitis and other typical demyelinating brain syndrome.^[5]^ ON is the first manifestation in 30% to 50% of NMOSD patients, and eventually involved optic nerve in 70% of patients.^[6–9]^ The idiopathic ON associated with NMO is called NMO-associated ON(NMO-ON), which is mainly prone to recurrence, poor prognosis and high rate of blindness,^[5]^ bringing heavy burdens on the life, work and study of patients, as well as the society and economy of various countries.^[1,10]^ Relevant clinical data show that after an average of 5 years of NMO, more than half of the patients will develop severe visual impairment in at least 1 eye.^[11]^ In particular, NMO-ON has poor recovery of visual impairment even after conventional treatment. They often develop into severe bilateral visual impairment in the long term, leaving behind varying degrees of optic atrophy, which is different from MS.^[12,13]^

Currently, there is no reference guideline for the treatment of NMO-ON. Recommendations for the treatment of NMO-ON attacks were adapted from studies of MS and idiopathic ON. Clinically, this class of drugs in treating NMO-ON is collectively referred to as disease modifying drugs,^[14]^ and the treatment is divided into 2 stages: acute phase and remission phase. The former is based on corticosteroids to reduce the severity of acute attacks. Treatment options include intravenous corticosteroids (IVCSs), plasma exchange (PLEX), and immunoglobulin. Immunosuppressive agents are often used in the latter to prevent recurrence and reduce the progression of neurological disability.^[15]^ Common drugs include mycophenolate, azathioprine, tacrolimus, and cyclosporine, etc.^[15]^ IVCSs is the first-line treatment options for NMOSD patients to reduce the severity of acute attacks but the effect is not very satisfactory.^[15]^ In particular, compared with the efficacy of other types of ON to IVCSs, NMO-ON has a worse response and poor efficacy.^[13,16]^ At the same time, there are contraindications and many potential complications in the use of IVCSs.^[17]^

Problems arise in patients who do not respond promptly to IVCS therapy or patients who are not suitable for IVCSs therapy, suggesting that more effective therapies are required. PLEX has been shown to be an effective treatment for NMO-ON due to the elimination of circulating pathogenic macromolecules such as AQP4-IgG, complement, or inflammatory cytokines, and the use of NMOSD therapy has increased year by year.^[18–20]^ It is traditionally believed that PLEX should be given as an additional treatment or salvage therapy for NMOSD patients with extremely severe disease, poor or ineffective IVCSs response.^[21]^ However, increasing scholars believe that delaying the time to start PLEX awaiting favorable outcome in response to corticosteroids is detrimental for the patient. PLEX may be a promising first line therapeutic approach in the management of severe attacks of NMOSD.^[22]^ For example, studies have shown that additional PLEX therapy rapidly improves the visual acuity of steroid-resistant seropositive AQP4 NMO-ON.^[23]^ A recent retrospective study also showed that PLEX was more effective than IVCSs in the acute phase of NMO-ON.^[24]^ In addition, A prospective, randomized, controlled pilot study showed that early treatment with PLEX should be encouraged especially in NMO-ON with a severe acute attack.^[25]^

Although PLEX is increasingly used in NMO-ON, its therapeutic effect and safety are still controversial. There are no systematic reviews yet that evaluated the effects of PLEX against other therapies in patients with NMO-ON. It is therefore timely to perform a systematic review to assess the efficacy and safety of PLEX on current research for its potential use in clinical practice in treating NMO-ON.

## 2 Methods

This protocol has been registered on PROSPERO (registration number: CRD42020162585).^[26]^ Our protocol will follow the Cochrane Handbook for Systematic Reviews of Interventions and the Preferred Reporting Items for Systematic Reviews and Meta-Analysis Protocol (PRISMA-P) statement guidelines.^[27,28]^

### 2.1 Inclusion criteria for study selection

#### 2.1.1 Types of studies

The systematic review will include all comparative researches, from randomized controlled trials (RCTs) to cohort studies, and case-control study. The current clinical trial results will be objectively integrated, which is conducive to the evaluation of the efficacy and safety of PLEX for NMO-ON. Uncontrolled trials, reviews, case studies, qualitative studies, animal trials, and laboratory studies will be excluded.

#### 2.1.2 Types of patients

Patients diagnosed as having NMO-ON will be included in the study. There will be no restrictions based on other conditions, such as age at onset, sex, ethnicity, educational or economic status, number of relapses prior to treatment, previous treatment, duration of illness, disease severity, and baseline expanded disability status scales (EDSS), AQP4-IgG serological status.

#### 2.1.3 Types of interventions

It was not restricted on the use of PLEX monotherapy alone. Patients in the experimental group were only treated with PLEX, or PLEX in combination with other therapies. Besides, the types, interval, number and frequency of PLEX were not limited. Studies that PLEX with combination therapy fail to objectively evaluate the efficacy and safety of PLEX will be excluded. The control interventions will include IVCSs, immunoglobulin, placebo, etc.

#### 2.1.4 Types of outcome measures

##### 2.1.4.1 Primary outcomes

1. Best-corrected visual acuity (BCVA): measured according to a validated measure such as the ETDRS chart, Snellen chart or a similar tool, other measures of visual acuity would be considered if outcomes could be justified and validated in relation to accepted relevant standard measures. Outcome measured was the mean change in the BCVA from before and after PLEX treatment.^[29]^
2. Annualized relapse rate (ARR): A relapse is defined as neurologic symptoms lasting for >24 hours, which occur at least 30 days after the onset of a preceding event. ARR is computed as a function of the number of relapse over the number of days (years) in observation. Post-treatment ARR were compared to pre-treatment ARR.^[30]^
3. The frequency and extent of Adverse events (AEs): Any symptomatic events which had a possible, probable, or definite causal relationship with PLEX treatment were defined as AEs during the treatment and follow-up periods (3 levels:^[31]^ mild, moderate, or severe. Briefly, “mild” AEs included those that were transient, had little or no clinical significance, and had no temporary interruption of any procedures. AEs, which required medical intervention and were not life-threatening, were classified as “moderate.” Potentially life-threatening events that required termination of the procedure were classified as “severe”).

##### 2.1.4.2 Secondary outcomes

1. EDSS: Disability progression was defined as an increase of at least 1 point above the pre-treatment score if baseline score < 5.5, and of at least a half point if baseline score > 5.5, of the Kurtzke EDSS. Outcome measured was the mean change in the EDSS from before and after PLEX treatment.^[32,33]^
2. Relapse-free rate: the absence of relapse during the observation period of the study reported as percentage per study.^[33]^
3. Peri-papillary retinal nerve fibers layer (pRNFL) or macular volume: change of the thickness of pRNFL or macular volume measured with optical coherence tomography (OCT) before and after PLEX treatment.
4. Visual electrophysiology examinations
5. Standard automated perimetry examinations
6. Time to the next attack.

##### 2.1.4.3 Security index

The safety was assessed by the occurrence of AEs. Any unexpected events that occurred during the studies will be recorded on an adverse event report form, including:

1. General physical examination (temperature, pulse, respiration, blood pressure).
2. Routine examination of blood, urine, and stool.
3. Liver and kidney function examination.
4. Electrocardiogram.
5. Possible AEs and related detection indicators.

### 2.2 Search methods for the identification of studies

#### 2.2.1 Electronic searches

A relevant literature search by sensitive search strategies was conducted using the following electronic databases from their inception to November 30, 2019: PubMed, Web of Science, EMBASE, the Cochrane Library, China National Knowledge Infrastructure (CNKI), Wanfang Database, China Science and Technology Journal database (VIP) and CBM. Search methods of MeSH terms with free words were applied in English databases. The related terms are as follows: Participants (neuromyelitis optica [MeSH], optic neuritis [MeSH], optic neuritis, neuromyelitis optica spectrum disorders), Intervention (plasma exchange [MeSH], plasma exchange, PE, PLEX, plasmapheresis [MeSH], plasmapheresis). The search strategy for PubMed is listed in Table 1, which including all search terms, and other searches will be conducted based on these results. This will be appropriately adapted for search in the other databases. There are no limits on language and publication status.

**Table 1:**
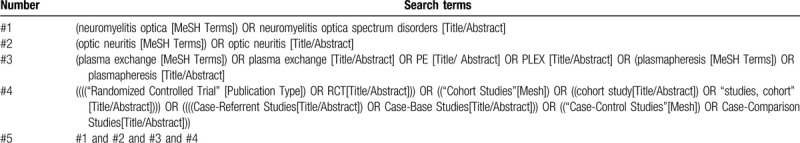
Search strategy used in PubMed database.

#### 2.2.2 Searching other resources

We will also search PROSPERO, the International Clinical Trials Registry Platform (ICTRP), ClinicalTrials.gov, dissertations, and gray literature to identify systematic reviews or clinical trials related to plasma exchange and NMO-ON. Relevant journals and conference processes will be manual searched. We will also review papers and bibliographies included in the trials.

### 2.3 Data collection and analysis

#### 2.3.1 Selection of studies

**Figure 1:**
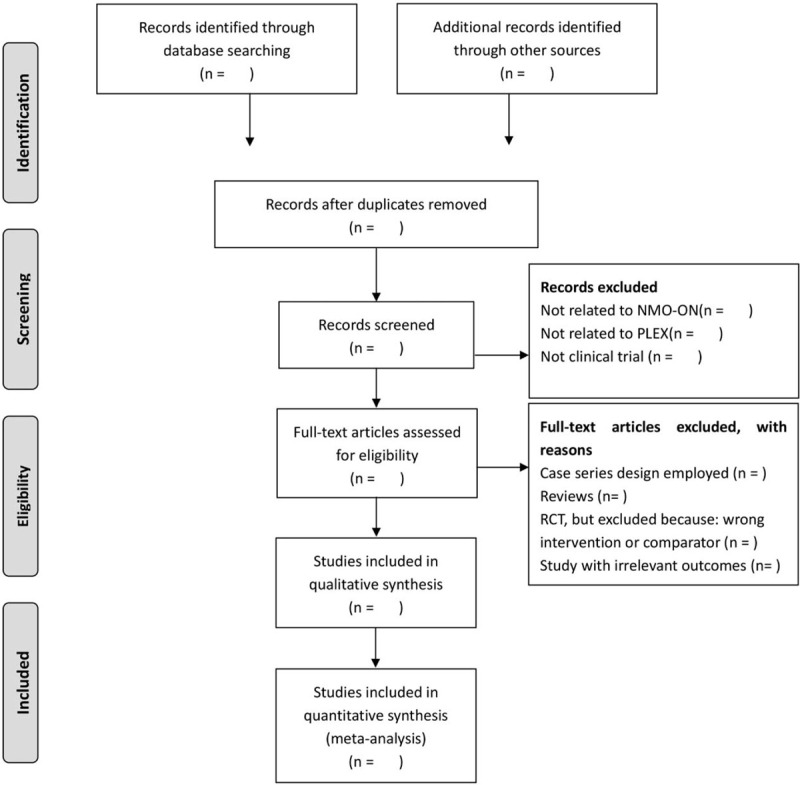
The PRISMA flow chart of the selection process. PRISMA = preferred reporting items for systematic reviews and meta-analysis protocol.

Two reviewers (MYH and LH) will independently browse the titles and abstracts of all of the retrieved records to distinguish and exclude any obviously irrelevant articles. We will select studies involved any form of PLEX as the sole treatment or as a major therapy. PLEX will be classed as the major therapy when the literature suggests that the frequency of application of PLEX is higher and the time is longer than other intervention methods. Studies only related to human subjects will be included. Any disagreements will be resolved by discussion between the 2 authors and an arbiter (ZJW). The study selection procedure is presented in a PRISMA flow chart (Fig. 1).

#### 2.3.2 Data extraction and management

Based on the inclusion criteria, a standard data collection form will be produced prior to data extraction. Search results will be entered into an EndNote X9 database and duplicate entries removed. Two authors (MYH and YC) will extract the data of interest from the eligible study and enter the following information in the data extraction sheet: The basic characteristics of each study (study type, author, title, source/journal, time of publication, country, hospital setting, study design); participants characteristics (average age, gender, sample size, inclusion and exclusion criteria, baseline situation); Interventions (type of PLEX, randomization, allocation concealment, blinding methods, and duration and frequency); Comparators (placebo, IVCSs, immunoglobulin); Outcomes (measures, main outcomes, security indexes, and follow up); If funded, it will also be recorded. When the consensus on data extraction is not available through discussion, the third reviewer (MJ) will make a decision.

#### 2.3.3 Assessment of risk of bias

Two authors (ZQL and LQN) will independently evaluate the risk and bias using the Cochrane risk of bias (ROB) assessment tool.^[34]^ The RevMan software program (V.5.3) will record the selected details of each study.^[35]^

#### 2.3.4 Measures of treatment effect

The risk ratio (RR) and 95% confidence interval (CI) will be used to analyze dichotomous data and measure the treatment effect. A weighted mean difference (WMD) or a standard mean difference (SMD) with 95% CIs will be used to analyze continuous outcomes.

#### 2.3.5 Unit of analysis issue

We will only extract the 1st experimental period data of crossover trials to avoid carryover effects. Meanwhile, considering that there are multiple intervention groups in trials, we will combine all analogous groups into a single pairwise comparison to prevent a unit of analysis issue.

#### 2.3.6 Management of missing data

Reviewer (ZQL) will contact the appropriate author of the included trials for clarification or more details via email and telephone if necessary. The missing data will be deleted, if there is no response from the author. In this case, this will be addressed in the discussion. Qualitative analysis would be used if relevant data was not available.

#### 2.3.7 Assessment of heterogeneity and data synthesis

We will use the complete case data as the analysis data. Heterogeneity will be tested with a standard Chi-Squared test.^[36]^ In order to quantify the impact of the statistical heterogeneity on the systematic review, the *I*^2^ value will be applied to calculate and present the heterogeneity degree. When *P* > .1, *I*^2^ < 50%, it is considered that there is no heterogeneity between the trials, and the fixed effect model will be used, otherwise, the random effect model will be adopted. All statistical analyses will be performed using RevMan5.3 software provided by the Cochrane Collaboration. Using the software to obtain forest plots and test the heterogeneity between the included studies. The Grades of Recommendation, Assessment, Development and Evaluation (GRADE) will be use to assess the meta-analysis findings and determine the quality of evidence. Narrative comprehensive synthesis will be adopted, if meta-analysis is not possible due to lack of clinical studies or heterogeneity.

#### 2.3.8 Assessment of reporting biases

When 10 or more studies are included in a meta-analysis, we will assess funnel plot asymmetry for reporting biases and small study effects using Egger method.^[37]^ For Egger test, *P* value of greater than .05 was determined as no considerable publication bias or small-study effects in studies. As funnel plot asymmetry does not necessarily suggest reporting bias, we will try to distinguish possible reasons for the asymmetry, including poor methodological quality and true heterogeneity of studies.

#### 2.3.9 Subgroup analysis

When heterogeneity is detected, a subgroup analysis will be conducted to judge the source of heterogeneity. The criteria for a subgroup analysis are as follows:

1. Type of PLEX.
2. Research quality.
3. Participation population.
4. Type of control interventions.
5. Intervention number, frequency, and duration.
6. AQP4-IgG serological status

#### 2.3.10 Sensitivity analysis

In the case of sufficient trials data, the ROB tool will be used to assess methodological quality. If low-quality articles are deleted, a second meta-analysis will be performed. The results and effect size of the 2 meta-analyses will be compared and discussed.^[38]^

## 3 Discussion

NMOSD is an inflammatory and heterogeneous astrocyte disorder of the CNS with the characteristic of higher incidence in women and Asian, concerned because of its high pathogenicity, high risk of recurrence, and poor prognosis.^[1]^ ON is the first manifestation in 30% to 50% of NMOSD patients, and eventually involved optic nerve in 70% of patients.^[6–9]^ NMO-ON is mainly prone to recurrence, poor prognosis and high rate of blindness,^[5]^ bringing heavy burdens on the life, work and study of patients, as well as the society and economy of various countries.^[1,10]^ At present, the treatment of NMO-ON is divided into 2 stages: acute phase (IVCSs, PLEX, and immunoglobulin) and remission phase (mycophenolate, azathioprine, tacrolimus, and cyclosporine, etc.).^[15]^ IVCSs is the first-line treatment options for NMO-ON patients to reduce the severity of acute attacks but the effect is not very satisfactory.^[15]^ In particular, compared with the efficacy of other types of ON to IVCSs, NMO-ON has a worse response and poor efficacy.^[13,16]^ At the same time, there are contraindications and many potential complications in the use of IVCSs.^[17]^ Problems arise in patients who do not respond promptly to IVCSs therapy and patients who are not suitable for IVCSs therapy, suggesting that more effective therapies are required.

PLEX has been shown to be an effective treatment for NMO-ON with fewer AEs and more therapeutic effects, due to the elimination of circulating pathogenic macromolecules such as AQP4-IgG, complement, or inflammatory cytokines.^[18–20]^ Emerging evidences have also proved the important value and potential prospect of PLEX in NMO-ON acute phase.^[21–25]^ However, there are controversial about its therapeutic effect and safety. It is therefore timely to perform a systematic review to assess the efficacy and safety of PLEX in treating NMO-ON on current research. The presented evidences were collected from RCTs with different evidence strengths to provide more comprehensive analysis. We expect that this systematic review will benefit patients with NMOSD, clinicians, healthcare managers, and policy-makers.

## Author contributions

MYH and YC conceived and designed the protocol, and MYH drafted the protocol manuscript. MYH developed the search strategy, with input from YC. MYH, LH and ZQL planned the data extraction. MYH, LQN and ZJW planned the quality appraisal of all included studies. MYH, LH, YC, ZQL, LQN and ZJW critically revised the manuscript for methodological and intellectual content. All authors approved the final version.

**Conceptualization:** Mengyu Han, Lu Hao, You Chen.

**Data curation:** Mengyu Han, Lu Hao, Ziqiang Liu, Luqi Nong.

**Formal analysis:** Mengyu Han, You Chen.

**Project administration:** Mengyu Han, Zhijun Wang.

**Supervision:** Mengyu Han, Zhijun Wang.

**Writing – review & editing:** Mengyu Han, You Chen.
